# Alzheimer-typical temporo-parietal atrophy and hypoperfusion are associated with a more significant cholinergic impairment in amnestic neurodegenerative syndromes

**DOI:** 10.1177/13872877251324080

**Published:** 2025-03-21

**Authors:** Nils Richter, Laura Breidenbach, Maximilian HT Schmieschek, Wolf-Dieter Heiss, Gereon R Fink, Oezguer A Onur

**Affiliations:** 1 Cognitive Neuroscience, Institute of Neuroscience and Medicine (INM-3), Research Center Jülich, Germany; 2 Department of Neurology, University Hospital Cologne and Faculty of Medicine, University of Cologne, Germany; 3 Max-Planck-Institute for Neurological Research, Cologne, Germany

**Keywords:** acetylcholinesterase, Alzheimer's disease, basal forebrain, dementia, hypometabolism, magnetic resonance imaging, mild cognitive impairment, MP4A, positron emission tomography, voxel-based morphometry

## Abstract

**Background:**

To date, cholinomimetics remain central in the pharmacotherapy of Alzheimer's disease (AD) dementia. However, postmortem investigations indicate that the AD-typical progressive amnestic syndrome may also result from predominantly limbic non-AD neuropathology such as TDP-43 proteinopathy and argyrophilic grain disease. Experimental evidence links a beneficial response to cholinomimetics in early AD to reduced markers of cholinergic neurotransmission. However, the cholinergic impairment varies among patients with a clinical AD presentation, likely due to non-AD (co)-pathologies.

**Objective:**

This study examines whether AD-typical atrophy and hypoperfusion can provide information about the cholinergic system in clinically diagnosed AD.

**Methods:**

Thirty-two patients with amnestic mild cognitive impairment or mild dementia due to AD underwent positron emission tomography (PET) with the tracer N-methyl-4-piperidyl-acetate (MP4A) to estimate acetylcholinesterase (AChE) activity, neurological examinations, cerebral magnetic resonance imaging (MRI) and neuropsychological assessment. The ‘cholinergic deficit’ was computed as the deviation of AChE activity from cognitively normal controls across the cerebral cortex and correlated gray matter (GM) and perfusion of temporo-parietal cortices typically affected by AD and basal forebrain (BF) GM.

**Results:**

Temporo-parietal perfusion and GM, as well as the inferior temporal to medial temporal ratio of perfusion correlated negatively with the ‘cholinergic deficit’. A smaller Ch4p area of the BF was associated with a more significant ‘cholinergic deficit’, albeit to a lesser degree than cortical measures.

**Conclusions:**

In clinically diagnosed AD, temporo-parietal GM and perfusion are more closely associated with the ‘cholinergic deficit’ than BF volumes, making them possible markers for cholinergic treatment response in amnestic neurodegeneration.

## Introduction

Alzheimer's disease (AD), biologically characterized by the accumulation of amyloid and tau pathologies and subsequent neurodegeneration,^1^ typically first presents with slowly progressive memory impairment. The observation of particularly severe degeneration of the cholinergic basal forebrain in AD led to the use of cholinomimetics, which to date are central to AD pharmacotherapy.^2,3^ However, the effects of these medications are limited and vary considerably across patients, while side-effects often limit their use. Nonetheless, cholinergic pharmacotherapy is likely to remain relevant despite the introduction of anti-amyloid medications, given the moderate effects, side-effects, and contraindications of current anti-amyloid therapies and the lack of alternatives.^4^ Given the enormous burden of AD and other amnestic neurodegenerative syndromes on patients and caregivers and the risk of side-effects in the often geriatric patients, it is therefore crucial to identify factors that will allow a targeted use of cholinergic medications and ensure an appropriate risk-benefit balance for each patient.

An explanation for the variable treatment response and limited group level effects of cholinomimetics may be, that a relevant ‘cholinergic deficit’ is required for patients to benefit from these medications and that the degree of cholinergic degeneration varies considerably across patients. Supporting this idea, we previously observed that even patients with mild cognitive impairment (MCI) due to AD may benefit from cholinergic stimulation, but the treatment response depended on the degree of cholinergic impairment.^5^ Interestingly, even in this relatively homogenous group, the cortical levels of acetylcholinesterase (AChE), a critical enzyme of cholinergic neurotransmission, varied substantially between patients.^6^ There is also evidence for more severe cholinergic impairment in patients with early-onset than late-onset AD.^6–8^

A cause of the variability in cholinergic degeneration and, consequently, response to cholinergic treatment could be the heterogeneity of underlying neuropathology. Trials examining the efficacy of cholinergic medications recruited patients based purely on a clinical AD diagnosis.^9–11^ However, it has become clear, that the AD-typical amnestic syndrome may also be caused by non-AD pathologies.^12^ Non-AD pathological change is common and can be observed as co-pathology in up to half of the patients with molecular evidence of AD-pathology, especially with increasing age.^12–14^ It remains unclear, how limbic predominant non-AD pathologies, such as limbic-predominant age-associated TDP-43 encephalopathy (LATE) and argyrophilic grain disease, affect the cholinergic system. Basal forebrain atrophy has been linked to amyloid and Lewy-body pathology rather than LATE.^15^ Furthermore, the basal forebrain does not appear particularly susceptible to the TDP-43 pathology observed in frontotemporal lobar degeneration,^16^ and total basal forebrain volumes measured using MRI did not differ between patients with pure-AD and pure LATE neuropathological change.^17^

Specific molecular markers of cholinergic neurotransmission, such as the activity of critical enzymes, transporters, and receptors, can be quantified *in vivo* using positron emission tomography (PET).^18–20^ However, these techniques are resource-intensive, and their use, hence, remains restricted to specialized centers, limiting their utility in large-scale studies of treatment response. However, AD and non-AD (co)-pathologies are associated with specific hypometabolism and atrophy patterns:^13,21–25^ The common non-AD pathologies predominantly affect the medial temporal lobe, whereas AD also affects lateral temporal and parietal cortices. Furthermore, the volume of basal forebrain structures, which are the source of cholinergic input to the cerebral cortex, can be assessed with MRI methods similar to those routinely used in the clinical setting.^6,26,27^ Therefore, these markers, thought to reflect different underlying pathologies, might be suitable for indirectly assessing the degree of cholinergic dysfunction *in vivo*.

Hence, we here examined putative structural (MRI) and metabolic (PET) imaging markers that could provide insights regarding the integrity of the cortical cholinergic system in patients with a clinical diagnosis of AD. Specifically, based on our prior work, we hypothesized (1) that the volume of the posterior basal forebrain (Ch4p region) would be more closely correlated with levels of cortical AChE activity than that of the whole basal forebrain. Furthermore, we hypothesized (2) that the inferior temporal gyrus to medial temporal (ITM) ratio of cerebral perfusion, which contrasts perfusion in areas of AD- and non-AD-degeneration,^13,21^ and perfusion of temporo-parietal cortices, serving as markers of AD-specific degeneration, would also be correlated with cortical AChE activity.

## Methods

### Patients

In this retrospective analysis, we included data from 32 patients with a clinical diagnosis of mild dementia^28^ or MCI due to AD,^29^ who had undergone PET with the tracer N-methyl-4-piperidyl-acetate (MP4A) to assess AChE activity at the Department of Neurology of the University Hospital of Cologne in the years from 1998 to 2003. Both diagnostic groups were included in the present analysis, as they are neighboring or even overlapping stages along the AD continuum. Furthermore, their discrimination is often difficult in the clinical setting, relying on the caregivers’ accounts and the patients’ complaints.

During the clinical work-up, patients underwent a comprehensive neuropsychological assessment covering verbal and visual memory, attention, cognitive flexibility, speech, and global cognition. To operationalize the amnestic neurodegenerative syndrome, only patients with predominant complaints of memory impairment, objectified in at least one test of episodic memory, were included. Patients were not included in the present analysis, if significant neurological or psychiatric diagnoses such as stroke, intracranial mass, epilepsy, Parkinson's disease or Lewy body disease were present.

Psychoactive medication such as cholinesterase inhibitors, antidepressants, or sedatives had been paused before PET scanning. This retrospective analysis of clinical data was approved by the ethics committee of the Medical Faculty of the University Hospital of Cologne (Nr. 22-1342-retro).

### PET imaging

MP4A was synthesized as described in previous publications.^30,31^ PET scanning was performed using an ECAT EXACT HR scanner (CTI/Siemens Knoxville, TN, USA) following a protocol described in Haense et al. (2012).^30^ AChE activity was estimated as the hydrolysis rate k_3_ of MP4A at the voxel level using a 3-parameter compartment model.^30,32^ PET data were processed as follows: (1) Affine co-registration of the sum of the first 10 min of each PET scan to the H_2_O PET template in Montreal Neurological Institute space included in the software package Statistical Parametric Mapping (SPM; http://fil.ion.ucl.ac.uk/spm/); (2) rigid-body co-registration of all subsequent frames to the sum of the first 10 min in standard space; (3) filtering of all frames with a Gaussian kernel (full width at half maximum = 8 mm), with restriction of the smoothing kernel to exclude areas of high signal contrast; (4) extraction of the reference kinetic curve from a standard putamen region of interest (ROI); and (5) estimation of k_3_ of MP4A at the voxel level as implemented in the software VINCI (version 4.20, Max-Planck Institute for Metabolism Research, Cologne, Germany).

The AChE reduction is not readily assessed using an individual ROI, since its’ degree and spatial pattern can vary considerably across patients.^6,7^ Therefore, to capture the AChE reduction in a single parameter with greater sensitivity than simply averaging across the whole cortex, each patient's AChE activity map was transformed to voxel-wise Z-scores relative to the AChE-maps of a control sample. This sample consisted of 18 cognitively normal older adults (7 female, 11 male; mean age 65.2 years, standard deviation 6.7 years, range 53–77 years) that had undergone a comprehensive neuropsychological examination to rule out relevant cognitive impairment and had provided written informed consent to the examination (cf. Richter et al., 2019^6^ and 2022^33^).

We operationalized the ‘cholinergic deficit’ for each patient as the number of voxels in the cerebral cortex and medial temporal lobe with Z < −2 multiplied by the average Z-score within those subthreshold voxels.^6^ For ease of interpretation the resulting values were transformed from negative to positive values, i.e., a greater numerical value reflects a greater ‘cholinergic deficit’. Since the distribution of the resulting values was highly non-normal, it was log-transformed for the subsequent analyses. To demonstrate the plausibility of this parameter, we also computed between cortical atrophy and perfusion measures with global cortical k_3_ and lobe-wise cortical k_3_.

Tracer uptake during the early period of PET scans mainly reflects cerebral perfusion, which is closely correlated with cerebral glucose metabolism.^33–35^ For each patient, the frames covering the 90–450 s window of each PET scan, were summed to generate a perfusion map, which was divided by the average uptake across the whole brain for this period to obtain standard uptake value ratios (SUVR), as this approach previously yielded the closest correlation with fluorodeoxyglucose PET.^33^

### Magnetic resonance imaging

Clinical T_1_-MRI scans with resolutions ranging from 1.0 mm isotropic resolution to a 1.0 mm in-plane resolution and a slice thickness of 2.5 mm were acquired using various MRI scanners at the University Hospital and outpatient clinics. To harmonize these images for subsequent analyses, synthetic high-resolution T_1_-images were generated from these original clinical T_1_-images using the tool SynthSR, which is part of the software package FreeSurfer (version 7.4.1).^36,37^

### Voxel-based morphometry

Gray matter were assessed in the framework of voxel-based morphometry (VBM) as implemented in the Statistical parametric mapping (SPM) toolbox CAT12.^38^ The synthetic T_1_-images were first segmented into tissue classes and spatially normalized into Montreal Neurological Institute (MNI) space using the high-dimensional registration algorithm DARTEL.^39^ The resulting gray matter maps were modulated only for the nonlinear normalization to account for global differences in head size modeled by the linear transform. Average voxel intensities were then extracted from the modulated normalized gray matter maps using masks in MNI space for the ROI described in the following.

### Regions of interest

The basal forebrain ROIs were defined using a cytoarchitectonic map in standard MNI space that differentiates between five subregions: the posterior NbM (nucleus basalis of Meynert; Ch4p), the anterior medial and intermediate NbM (Ch4a-i), the anterior lateral NbM (Ch4al), the horizontal limb of the diagonal band of Broca (Ch3), and a subregion combining the vertical limb of the diagonal band of Broca and the medial septal nuclei (Ch2 and Ch1).^27^ A ROI for the whole basal forebrain was defined by summing up all of the subnuclei, and the posterior NbM (Ch4p) mask was used to extract gray matter from the Ch4p area.

A temporo-parietal meta region for AD-typical hypometabolism^22^ was generated by combining the angular gyrus, the posterior cingulate cortex, and the inferior temporal gyrus from the Harvard-Oxford-Atlas.^40^ Furthermore, the ratio of glucose metabolism between the inferior temporal gyrus and the medial temporal lobe, which distinguishes between AD and non-AD limbic pathologies,^13,21^ was computed for cerebral perfusion and gray matter. The respective ROIs for the inferior temporal gyrus and the medial temporal lobe (amygdala and hippocampus) were defined using the Harvard-Oxford-Atlas, and the ROI means for perfusion and gray matter for the inferior temporal gyrus were divided by those for the medial temporal lobe ROI.

### Statistical analyses

Statistical analyses were performed using *Jeffreys’ Amazing Statistics Program* (JASP Version 0.18.3, jasp-stats.org). Correlations were assessed using Pearson's correlation coefficients bootstrapped with 1000 permutations, and results are presented with 95%-confidence intervals and two-sided p-values. All correlations were primarily computed for MCI and mild dementia patients combined since these two stages reflect adjacent diagnostic categories along the clinical spectrum. However, to rule out that group differences drove correlations, all correlations were also computed for the dementia patients only. Given the limited sample size (n = 6) and statistical power, the correlations were not computed separately for the MCI group.

Neither the ‘cholinergic deficit’ nor any ROI values tested for correlations with the ‘cholinergic deficit’ significantly correlated with age. Furthermore, there was no difference between women and men in any of the parameters above, as assessed using independent samples T-tests. Means are presented with standard deviations (SD).

## Results

### Demographics

Thirty-two patients (20 female, 12 male) were included in the current analysis. The average age was 64.2 years (SD 8.7 years, range: 50–75 years). A MMSE score was available for 22 participants, with a mean MMSE of 23.9 (SD 2.9, range: 19–28). Clinical diagnoses were mild dementia in 26 patients and MCI in six patients.

### Cortical k_3_ and the ‘cholinergic deficit’

**Figure 1.**
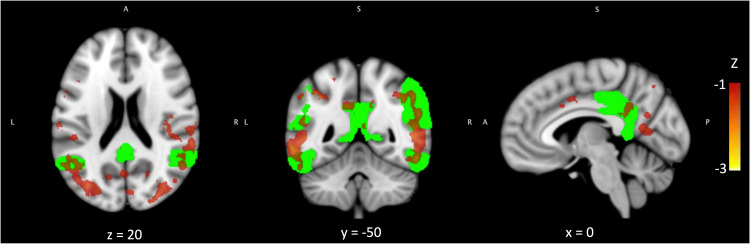
The ‘cholinergic deficit’ partially overlaps with the AD meta ROI.

The mean k_3_ of MP4A across the entire cerebral cortex was 0.075 (SD 0.013). k_3_ was highest in the frontal lobe (mean 0.083, SD 0.013) and lowest in the parietal lobe (mean 0.066, SD 0.012). Mean ROI values for gray matter, k_3_, and perfusion are summarized in Table 1. The ‘cholinergic deficit’ was most prominent in the lateral temporal and parietal lobes, extending into the occipital lobe but mostly sparing the parietal midline structures. It partially overlapped with the temporo-parietal AD meta region, particularly in the inferior temporal and angular gyri (Figure 1).

The group average of the ‘cholinergic deficit’ (red to yellow) superimposed on the AD meta ROI (green). The two partially overlap in lateral temporal and parietal areas, but the ‘cholinergic deficit’ also extends to the occipital lobe while largely sparing the posterior cingulate and precuneus. x, y, and z denote slice coordinates in Montreal Neurological Institute space. ‘cholinergic deficit’ - number of voxels, where acetylcholinesterase activity deviated more than two standard deviations below the mean of cognitively normal individuals, multiplied with the average Z-value of these subthreshold voxels. Z = Z-statistics of acetylcholinesterase activity relative to a reference sample of cognitively normal older adults. AD: Alzheimer's disease; ROI: region of interest. For visualization purposes the scale was set to Z = −1 to −3.

**Table 1.** Summary of imaging measures.

		Mean	SD	Minimum	Maximum
Acetylcholinesterase activity [k_3_]	Total Cortex	0.075	0.013	0.048	0.097
	Frontal Lobe	0.083	0.013	0.053	0.104
	Temporal Lobe	0.074	0.017	0.043	0.103
	Parietal Lobe	0.066	0.012	0.043	0.085
	Occipital Lobe	0.067	0.012	0.044	0.1
	'Cholinergic deficit'	30,784.922	46,900.401	410.698	148,512.07
	Log of the ‘cholinergic deficit'	3.983	0.739	2.614	5.172
Perfusion [SUVR]	Alzheimer's disease meta ROI	0.91	0.058	0.809	1.01
	Inferior temporal gyrus to medial temporal ratio	0.83	0.064	0.685	0.928
Gray matter [relative units]	Alzheimer's disease meta ROI	0.598	0.034	0.521	0.679
	Inferior temporal gyrus to medial temporal ratio	0.819	0.057	0.709	0.942
	Ch4p area of the basal forebrain	0.724	0.042	0.637	0.833
	Whole basal forebrain	0.776	0.06	0.648	0.92

SD: standard deviation; SUVR: standardized uptake value ratio; ROI: region of interest; ‘cholinergic deficit’: number of voxels, where acetylcholinesterase activity deviated more than two standard deviations below the mean of cognitively normal individuals, multiplied with the average Z-value of these subthreshold voxels.

### The ‘cholinergic deficit’ correlated negatively with posterior basal forebrain volume

**Figure 2.**
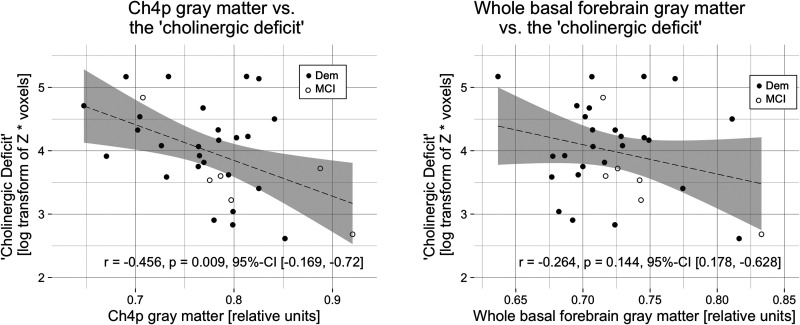
The ‘cholinergic deficit’ correlated negatively with posterior basal forebrain gray matter.

The extent of ‘cholinergic deficit’ was negatively correlated with the volume of the Ch4p region of the basal forebrain (r = −0.456, p = 0.009, 95%-CI [−0.169, −0.72]), but not with the volume of the whole basal forebrain (r = −0.264, p = 0.144, 95%-CI [0.178, −0.628]; Figure 2).

The gray matter of the Ch4p area correlated negatively with the ‘cholinergic deficit’, whereas the gray matter of the whole basal forebrain did not. The Pearson correlation coefficient r is reported with bootstrapped 95%-confidence intervals and 2-sided p-values. ‘cholinergic deficit’ - number of voxels, where acetylcholinesterase activity deviated more than two standard deviations below the mean of cognitively normal individuals, multiplied with the average Z-value of these subthreshold voxels. Dem: dementia; MCI: mild cognitive impairment.

### The ‘cholinergic deficit’ was associated with AD-typical temporo-parietal hypoperfusion

**Figure 3.**
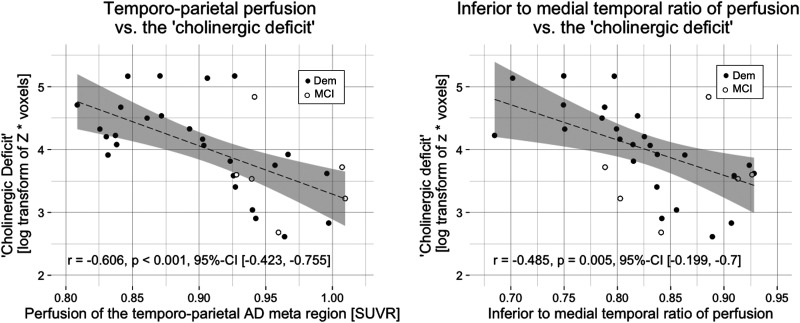
The ‘cholinergic deficit’ correlated negatively with AD-typical hypoperfusion.

A strong negative correlation was observed between the ‘cholinergic deficit’ and perfusion in the temporo-parietal AD meta region (r = −0.606, p < 0.001, 95%-CI [−0.423, −0.755]). Furthermore, the ITM ratio of perfusion was also negatively correlated with the ‘cholinergic deficit’ (r = −0.485, p = 0.005, 95%-CI [−0.199, −0.7]; Figure 3).

Cerebral perfusion in the temporo-parietal AD meta region and the inferior to medial temporal ratio of cerebral perfusion correlated negatively with the ‘cholinergic deficit’. The temporo-parietal AD meta region consisted of the inferior temporal gyrus, the angular gyrus and the posterior cingulate cortex. The Pearson correlation coefficient r is reported with bootstrapped 95%-confidence intervals and 2-sided p-values. ‘cholinergic deficit’: number of voxels, where acetylcholinesterase activity deviated more than two standard deviations below the mean of cognitively normal individuals, multiplied with the average Z-value of these subthreshold voxels; AD: Alzheimer's disease; Dem: dementia; MCI: mild cognitive impairment; SUVR: standardized uptake value ratio.

### Temporo-parietal gray matter correlated negatively with the ‘cholinergic deficit’

**Figure 4.**
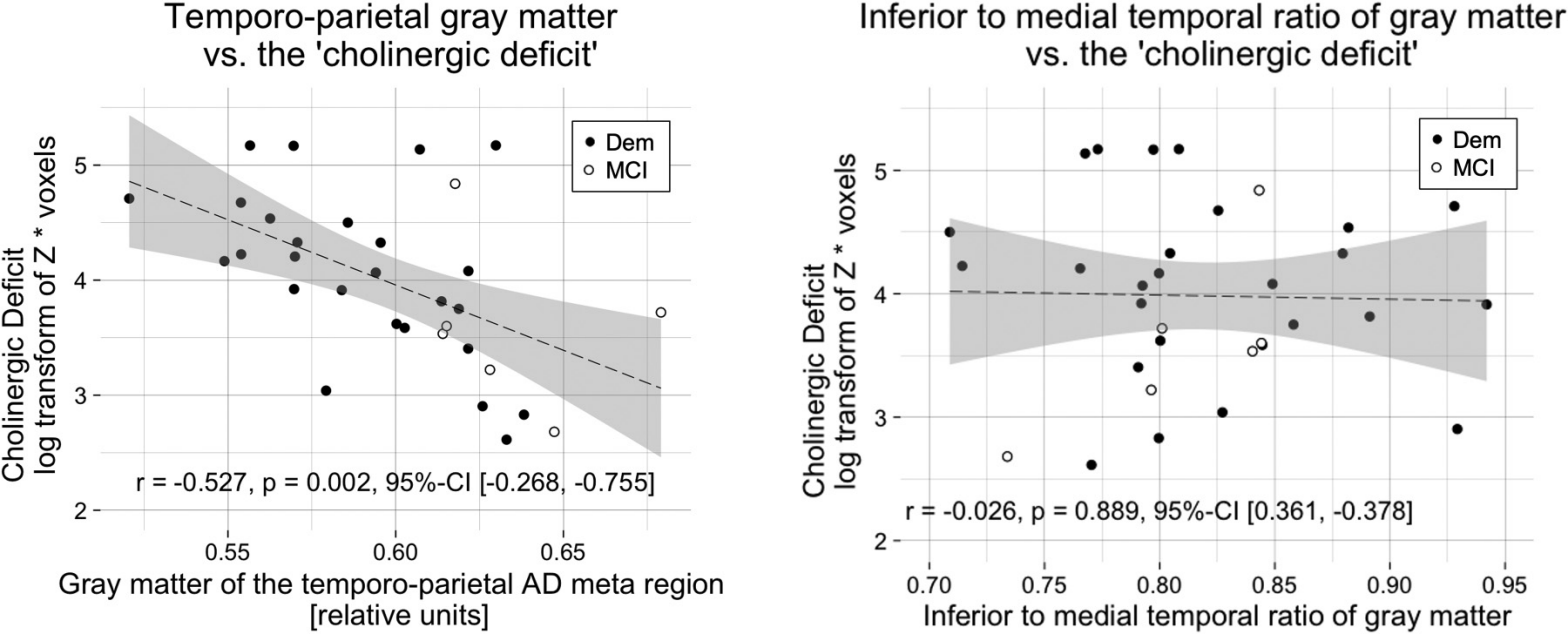
The ‘cholinergic deficit’ correlated negatively with temporo-parietal gray matter.

Further, we explored whether the association between AD-typical perfusion and the ‘cholinergic deficit’ could also be translated to gray matter in the temporo-parietal AD meta region or the ITM ratio of gray matter. The gray matter of the temporo-parietal AD meta region correlated negatively with the ‘cholinergic deficit’ (r = −0.527, p = 0.002, 95%-CI [−0.268, −0.755]), while the ITM ratio of gray matter did not (r = −0.026, p = 0.889, 95%-CI [0.361, −0.378]; Figure 4).

Gray matter of the temporo-parietal AD meta region, but not the inferior to medial temporal ratio of gray matter, correlated negatively with the ‘cholinergic deficit’. The temporo-parietal AD meta region consisted of the inferior temporal gyrus, the angular gyrus and the posterior cingulate cortex. The Pearson correlation coefficient r is reported with bootstrapped 95%-confidence intervals and 2-sided p-values. ‘cholinergic deficit’: number of voxels, where acetylcholinesterase activity deviated more than two standard deviations below the mean of cognitively normal individuals, multiplied with the average Z-value of these subthreshold voxels; AD: Alzheimer's disease; Dem: dementia; MCI: mild cognitive impairment.

### Associations between the ‘cholinergic deficit’ and imaging markers in dementia

To rule out that the observed correlations were merely attributable to differences in the measures between MCI and dementia patients we also performed the correlation analyses separately for the dementia group, excluding the six MCI patients. The correlations between the ‘cholinergic deficit’ and the perfusion measures as well as the gray matter within the temporo-parietal AD meta region remained significant. In contrast, the association with Ch4p volume was not present within the dementia group alone (Table 2).

**Table 2.** Correlations of gray matter and perfusion with the ‘cholinergic deficit’ in the mild dementia subgroup.

	Ch4p	Whole BF	Perfusion of AD meta ROI	ITM ratio of perfusion	GM of AD meta ROI	ITM ratio of GM
Pearson's r	−0.319	−0.1	−0.613	−0.585	−0.515	−0.155
p	0.112	0.627	**<0.001**	**0**.**002**	**0**.**007**	0.448
Upper 95% CI	0.085	0.443	−0.357	−0.28	−0.112	0.216
Lower 95% CI	−0.648	−0.528	−0.792	−0.828	−0.803	−0.47

Correlations between the ‘cholinergic deficit’ and the different ROI values only in the 26 patients with dementia. In this subgroup, only the two perfusion measures and gray matter in the temporo-parietal AD meta region correlated significantly with the ‘cholinergic deficit’. AD: Alzheimer's disease; BF: basal forebrain; GM: gray matter; ROI: region of interest; ITM: inferior temporal gyrus to medial temporal lobe; CI: confidence interval. Bold font indicates statistical significance at a two-sided p < 0.05. ‘cholinergic deficit’ - number of voxels, where acetylcholinesterase activity deviated more than two standard deviations below the mean of cognitively normal individuals, multiplied with the average Z-value of these subthreshold voxels.

## Cortical AChE activity correlated positively with temporo-parietal GM and perfusion

A sensitivity analysis demonstrated that cortical AChE activity, especially of the temporal, parietal and occipital cortices, was positively correlated with GM and perfusion in the temporo-parietal AD meta region, inferior to medial temporal ratio of perfusion, and GM of the posterior basal forebrain (Ch4p area; Table 3, Supplementary Figures 1–3).

**Table 3.** Cortical AChE activity is correlated with cerebral perfusion and gray matter.

Whole cortex mean acetylcholinesterase activity [k3]					
		r	p	upper 95% CI	lower 95% CI
Perfusion [SUVR]	AD meta ROI	0.482	**0**.**005**	0.662	0.233
	ITM ratio	0.493	**0**.**004**	0.745	0.15
Gray matter [relative units]	AD meta ROI	0.418	**0**.**017**	0.657	0.143
	ITM ratio	0.043	0.815	0.317	−0.27
	Ch4p	0.381	**0**.**032**	0.657	0.062
	Whole basal forebrain	0.256	0.157	0.597	−0.204
Frontal cortex acetylcholinesterase activity [k3]					
		r	p	upper 95% CI	lower 95% CI
Perfusion [SUVR]	AD meta ROI	0.271	0.133	0.508	−0.009
	ITM ratio	0.408	**0**.**02**	0.717	−0.026
Gray matter [relative units]	AD meta ROI	0.238	0.189	0.491	−0.04
	ITM ratio	0.174	0.342	0.414	−0.089
	Ch4p	0.198	0.277	0.499	−0.125
	Whole basal forebrain	0.132	0.472	0.489	−0.295
Temporal cortex acetylcholinesterase activity [k3]					
		r	p	upper 95% CI	lower 95% CI
Perfusion [SUVR]	AD meta ROI	0.672	**<0**.**001**	0.811	0.475
	ITM ratio	0.513	**0**.**003**	0.751	0.22
Gray matter [relative units]	AD meta ROI	0.588	**<0**.**001**	0.773	0.338
	ITM ratio	−0.033	0.856	0.249	−0.351
	Ch4p	0.507	**0**.**003**	0.726	0.226
	Whole basal forebrain	0.334	0.062	0.637	−0.081
Parietal cortex acetylcholinesterase activity [k3]					
		r	p	upper 95% CI	lower 95% CI
Perfusion [SUVR]	AD meta ROI	0.502	**0**.**003**	0.682	0.261
	ITM ratio	0.48	**0**.**005**	0.748	0.153
Gray matter [relative units]	AD meta ROI	0.425	**0**.**015**	0.681	0.112
	ITM ratio	0.01	0.958	0.307	−0.33
	Ch4p	0.413	**0**.**019**	0.688	0.078
	Whole basal forebrain	0.247	0.173	0.577	−0.206
Occipital cortex acetylcholinesterase activity [k3]					
		r	p	upper 95% CI	lower 95% CI
Perfusion [SUVR]	AD meta ROI	0.505	**0**.**003**	0.689	0.304
	ITM ratio	0.555	**<0**.**001**	0.73	0.31
Gray matter [relative units]	AD meta ROI	0.42	**0**.**017**	0.658	0.156
	ITM ratio	−0.099	0.591	0.234	−0.464
	Ch4p	0.401	**0**.**023**	0.717	0.045
	Whole basal forebrain	0.289	0.109	0.612	−0.172

AD meta ROI: inferior temporal gyrus, angular gyrus and posterior cingulate cortex; CI: confidence interval; ITM: inferior temporal gyrus to medial temporal lobe. The Pearson correlation coefficient r is reported with bootstrapped 95%-confidence intervals and 2-sided p-values. Bold font indicates statistical significance at a two-sided p < 0.05.

## Discussion

The current data indicate that greater AD-typical temporo-parietal hypometabolism is associated with a more significant ‘cholinergic deficit’ in patients presenting with a clinical AD phenotype, i.e., an amnestic neurodegenerative syndrome. Gray matter of the same cortical areas was also negatively correlated with the ‘cholinergic deficit’, even more so than the gray matter of the posterior N. basalis Meynert (Ch4p). The whole basal forebrain volume, however, was not correlated with the ‘cholinergic deficit’.

Cortical hypometabolism and gray matter atrophy thus appear to be more closely related to the ‘cholinergic deficit’ than the volumes of the whole cholinergic basal forebrain or the Ch4p subregion. Similarly, cortical AChE activity, especially in the temporal and parietal cortex is positively correlated with temporo-parietal GM and perfusion, while showing a weaker association with Ch4p volume. These associations are unlikely to be a simple consequence of cortical hypometabolism or atrophy and consecutive loss of local cholinergic input because the ‘cholinergic deficit’ in this sample, as previously described in MCI due to AD,^33^ mainly affects the lateral temporal and parietal cortices, only partially overlapping with the posterior midline structures usually most severely affected in AD^23^ (Figure 1). Furthermore, the presently observed predominantly lateral distribution of the ‘cholinergic deficit’ is in line with the anatomy of the cholinergic system, as the posterior part of the basal forebrain that is predominantly affected in AD mainly provides cholinergic projections to the lateral temporal and parietal lobe.^41–43^ This, however, raises the question of why the cortical neurodegeneration markers appear more closely linked to the ‘cholinergic deficit’ than the volume of the Ch4p area. An explanation may lie in the small size of the basal forebrain nuclei, which are on the same order of magnitude as the resolution of the used MR scans. Another explanation may lie in the structure of the Ch4 area: It lacks distinct anatomical boundaries with a degree of overlap between the subnuclei,^41,42^ which may not be fully captured using structural MRI. On the other hand, the fact that Ch4p volume was weakly correlated with the ‘cholinergic deficit’, while total basal forebrain volume was not, could indicate that the present MRI method did detect an association between Ch4p atrophy and the cortical cholinergic integrity, albeit with suboptimal sensitivity. After all, investigations applying similar methods indicate that structural MRI can capture differential associations of the basal forebrain subnuclei with molecular markers of cholinergic neurotransmission.^6,44^ Furthermore, the differential association of the Ch4p area but not the whole basal forebrain with the ‘cholinergic deficit’ in the present data is corroborated by the observation that total basal forebrain volume does not differ between patients with pure AD pathology and patients with TDP-43 pathology in post mortem evaluations.^17^

The strong association of the ‘cholinergic deficit’ with perfusion and gray matter in the temporo-parietal AD meta region, which consists of the inferior temporal and angular gyri as well as the posterior cingulate cortex,^22^ implies, that cortical AD-typical neurodegeneration^1^ is the most suitable marker for the ‘cholinergic deficit’ in amnestic neurodegeneration investigated here. We also observed an association between the ITM ratio of cerebral perfusion as a proxy for glucose metabolism. This could serve as a further clue regarding the association of the ‘cholinergic deficit’ with different etiologies, as the ITM ratio of glucose metabolism has previously been demonstrated to discriminate between AD and non-AD pathologies.^13,21^ Hence, patients with predominantly non-AD pathology such as limbic predominant TDP-43, primary age-related tauopathy, or argyrophilic grain disease would be expected to have a smaller ‘cholinergic deficit’ than those with purer AD pathology.

While previous studies also reported the ITM ratio of gray matter to discriminate between AD and non-AD amnestic neurodegeneration,^17,25^ we did not observe an association between the ITM ratio of gray matter and the ‘cholinergic deficit’. However, the discriminative power of the ITM ratio of gray matter in those studies was lower than that previously reported for hypometabolism.^13,21^ Furthermore, Botha et al. (2018)^21^ also reported that, in contrast to the ITM ratio of glucose metabolism, inferior to medial temporal gray matter thickness did not differ between AD and non-AD amnestic pathologies, which may explain the absence of an association between the ITM of gray matter and the ‘cholinergic deficit’ observed here.

The utility of the ‘cholinergic deficit’ and cortical AD-typical neurodegeneration as predictors of the response to cholinergic pharmacotherapy needs to be systematically examined in adequately powered placebo-controlled investigations under ‘real-world’ conditions. The cortical AChE-activity used to quantify the ‘cholinergic deficit’ here has previously been linked to the response to a single dose of the cholinomimetic rivastigmine under controlled conditions.^5^ However, the findings from that study cannot be translated directly to the current observations, as that study only included AD biomarker positive patients at the stage of MCI, while current sample also included patients with mild dementia and unknown CSF status, i.e., a much more diverse sample. A promising observation, however, comes from a clinical trial examining predictors of the response to the AChE-inhibitor donepezil. In that study, patients with a hippocampal sparing subtype benefitted from the treatment, whereas those with limbic involvement did not.^45^ A confirmation of a smaller benefit from cholinergic pharmacotherapy in patients with less AD-typical cortical neurodegeneration would have great clinical relevance, as these patients are typically older^12,13,46^ and hence more prone to the side-effects of cholinomimetics, be it because of comorbidities, greater frailty, or interactions with co-medications.^47,48^

### Limitations

This investigation is limited by the sample size and its retrospective observational nature. Furthermore, our sample was relatively young with a mean age of 64.2 years. Hence, the amount of the age-associated limbic non-AD (co)-pathologies may have been lower than in other clinical samples. However, post mortem data indicate that relevant limbic TDP-43 pathology alone is observed in over 15% of patients younger than 75 years and that number steadily increases with age.^12,49^ Limbic non-AD (co-) pathologies are, therefore, likely to have played a role in the present sample. However, the observed effects could actually be more pronounced in a sample of older subjects, where AD pathology is less dominant. Furthermore, while not the focus of the present investigation, CSF biomarkers of AD-pathology could have provided additional context and permitted complementary analyses, e.g., regarding the strength of the reported associations in biomarker-positive and -negative individuals. However, CSF biomarker data was only available for a small subset of patients and different assays were employed, making it inadequate for meaningful analyses.

### Conclusions

In patients with a clinical AD phenotype, measures of AD-typical cortical neurodegeneration were more closely linked to the ‘cholinergic deficit’ than atrophy of the cholinergic basal forebrain. Hence, data suggest that imaging markers of this AD-typical degeneration may serve as predictors of the response to cholinergic pharmacotherapy in patients with amnestic neurodegenerative syndromes, be it due to AD or non-AD pathologies. More precise means to guide therapy are direly needed to effectively treat this steadily growing and aging patient group.

## Supplemental Material

sj-docx-1-alz-10.1177_13872877251324080 - Supplemental material for Alzheimer-typical temporo-parietal atrophy and hypoperfusion are associated with a more significant cholinergic impairment in amnestic neurodegenerative syndromes Supplemental material, sj-docx-1-alz-10.1177_13872877251324080 for Alzheimer-typical temporo-parietal atrophy and hypoperfusion are associated with a more significant cholinergic impairment in amnestic neurodegenerative syndromes by Nils Richter, Laura Breidenbach, Maximilian HT Schmieschek, Wolf-Dieter Heiss, Gereon R Fink and Oezguer A Onur in Journal of Alzheimer's Disease
