# Supplementary material for: Alzheimer-typical temporo-parietal atrophy and hypoperfusion are associated with a more significant cholinergic impairment in amnestic neurodegenerative syndromes
Source: J Alzheimers Dis. 2025 Mar 21;104(4):1290–300. doi: 10.1177/13872877251324080 (PMC12231882; doi:10.1177/13872877251324080)
Supplement: sj-docx-1-alz-10.1177_13872877251324080 - Supplemental material for Alzheimer-typical temporo-parietal atrophy and hypoperfusion are associated with a more significant cholinergic impairment in amnestic neurodegenerative syndromes [file sj-docx-1-alz-10.1177_13872877251324080.docx]

**Supplemental Material**

**Alzheimer-typical temporo-parietal atrophy and hypoperfusion are associated with a more significant cholinergic impairment in amnestic neurodegenerative syndromes**

**Supplemental Figure 1.** Cortical acetylcholinesterase activity correlated positively with posterior basal forebrain gray matter


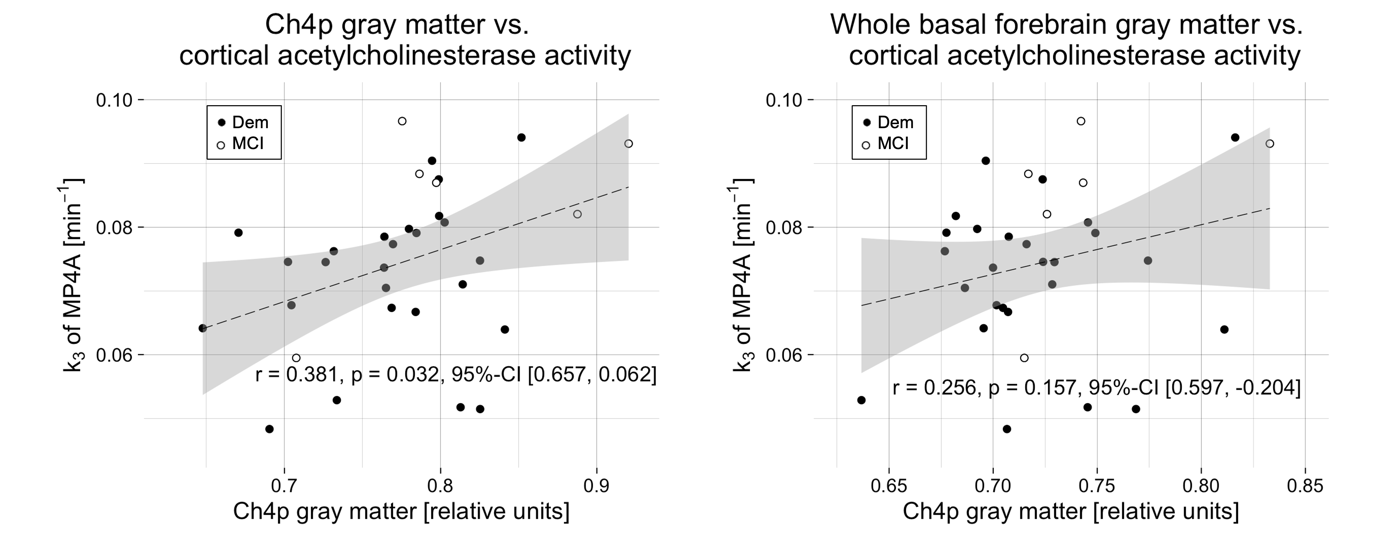


Gray matter of the Ch4p area correlated positively with acetylcholinesterase activity averaged across the whole cerebral cortex, while whole basal forebrain gray matter did not. The Pearson correlation coefficient r is reported with bootstrapped 95%-confidence intervals and 2-sided p-values. CI: confidence interval; Dem: dementia; MCI: mild cognitive impairment; MP4A: N-methyl-4-piperidyl-acetate.

**Supplemental Figure 2.** Cortical acetylcholinesterase activity correlated positively with temporo-parietal perfusion and the inferior to medial temporal ratio of perfusion.


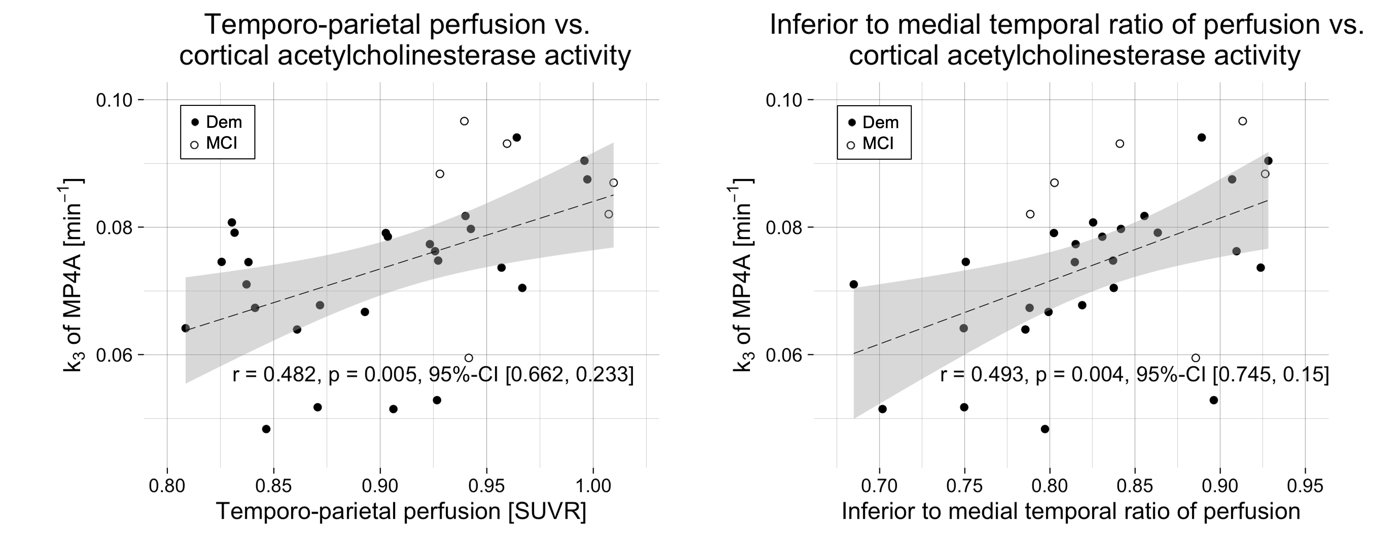


Cerebral perfusion of the temporo-parietal AD meta region and the inferior to medial temporal ratio of cerebral perfusion correlated positively with acetylcholinesterase activity averaged across the whole cerebral cortex. The temporo-parietal region of interest consisted of the inferior temporal gyrus, the angular gyrus and the posterior cingulate cortex. The Pearson correlation coefficient r is reported with bootstrapped 95%-confidence intervals and 2-sided p-values. AD: Alzheimer’s disease; CI: confidence interval; Dem: dementia; MCI: mild cognitive impairment; MP4A: N-methyl-4-piperidyl-acetate; SUVR: standardized uptake value ratio.

**Supplemental Figure 3.** Cortical acetylcholinesterase activity correlated positively with temporo-parietal gray matter


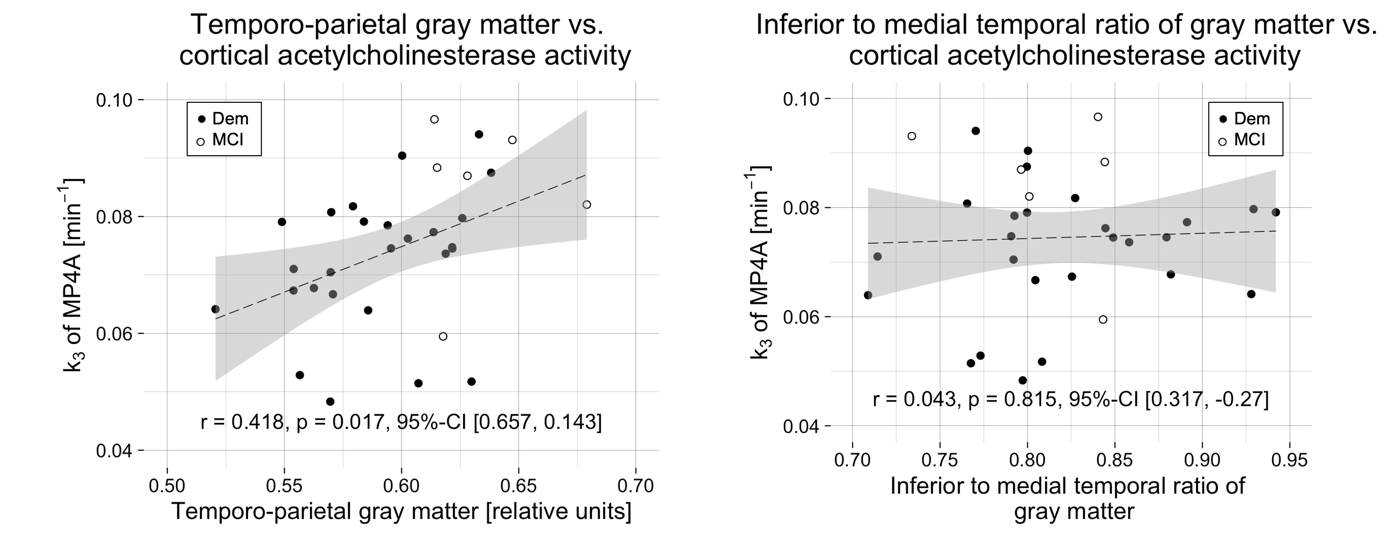


Gray matter of the temporo-parietal AD meta region, but not the inferior to medial temporal ratio of gray matter, correlated positively with acetylcholinesterase activity averaged across the whole cerebral cortex. The temporo-parietal region of interest consisted of the inferior temporal gyrus, the angular gyrus and the posterior cingulate cortex. The Pearson correlation coefficient r is reported with bootstrapped 95%-confidence intervals and 2-sided p-values. AD: Alzheimer’s disease; CI: confidence interval; Dem: dementia; MCI: mild cognitive impairment; MP4A: N-methyl-4-piperidyl-acetate.
